# MT1G serves as a tumor suppressor in hepatocellular carcinoma by interacting with p53

**DOI:** 10.1038/s41389-019-0176-5

**Published:** 2019-11-15

**Authors:** Yingchao Wang, Gaoxiong Wang, Xionghong Tan, Kun Ke, Bixing Zhao, Niangmei Cheng, Yuan Dang, Naishun Liao, Fei Wang, Xiaoyuan Zheng, Qin Li, Xiaolong Liu, Jingfeng Liu

**Affiliations:** 1 The United Innovation of Mengchao Hepatobiliary Technology Key Laboratory of Fujian Province, Mengchao Hepatobiliary Hospital of Fujian Medical University, Fuzhou, 350025 People’s Republic of China; 2 Department of Hepatobiliary Surgery, The Second Affiliated Hospital of Fujian Medical University, Quanzhou, 362001 People’s Republic of China; 3 The Liver Center of Fujian Province, Fujian Medical University, Fuzhou, 350025 People’s Republic of China; 4 Fujian Institute of Research on the Structure of Matter, Chinese Academy of Sciences, Fuzhou, Fujian 350002 People’s Republic of China; 5 Department of Comparative Medicine, Dongfang Affiliated Hospital of Xiamen University (900 Hospital of The Joint Logistics Team), Fuzhou, Fujian 350025 People’s Republic of China; 6 Department of Infectious Diseases, Mengchao Hepatobiliary Hospital of Fujian Medical University, Fuzhou, 350025 People’s Republic of China; 7 Liver Disease Center, The First Affiliated Hospital of Fujian Medical University, Fuzhou, 350005 People’s Republic of China

**Keywords:** Liver cancer, Cell growth

## Abstract

Poor prognosis of hepatocellular carcinoma (HCC) patients is frequently associated with rapid tumor growth, recurrence and drug resistance. MT1G is a low-molecular weight protein with high affinity for zinc ions. In the present study, we investigated the expression of MT1G, analyzed clinical significance of MT1G, and we observed the effects of MT1G overexpression on proliferation and apoptosis of HCC cell lines in vitro and in vivo. Our results revealed that MT1G was significantly downregulated in tumor tissues, and could inhibit the proliferation as well as enhance the apoptosis of HCC cells. The mechanism study suggested that MT1G increased the stability of p53 by inhibiting the expression of its ubiquitination factor, MDM2. Furthermore, MT1G also could enhance the transcriptional activity of p53 through direct interacting with p53 and providing appropriate zinc ions to p53. The modulation of MT1G on p53 resulted in upregulation of p21 and Bax, which leads cell cycle arrest and apoptosis, respectively. Our in vivo assay further confirmed that MT1G could suppress HCC tumor growth in nude mice. Overall, this is the first report on the interaction between MT1G and p53, and adequately uncover a new HCC suppressor which might have therapeutic values by diminishing the aggressiveness of HCC cells.

## Introduction

Hepatocellular carcinoma (HCC) is the sixth most common cancer worldwide and the third most common cause of cancer-related death in the Asia-Pacific region^1^. Although surgical resection combined with radio- and chemotherapy is potentially curative in some cases, patients with advanced HCC show poor outcomes^2–4^, which probably is attributed to HCC rapid growth, drug resistance and metastasis after surgical excision. Hence, the identification of new carcinogenic proteins or inhibitors of HCC is necessary to further understand the progression mechanism of HCC and broaden the potential of targeted therapy.

Metallothioneins (MTs) are a family of low-molecular weight, cysteine-rich intracellular proteins, that play critical roles in antioxidants and preserve homeostasis of biologically essential metals, such as zinc and copper^5,6^. Given their capacity to influence metabolism of metals, MTs might also regulate the function of zinc-dependent proteins, such as p53 (ref. ^7^), transcription factor, and chemo-sensitivity, by donating or taking away of zinc ions. Hence, the expression of MTs is also widely associated with aggressive phenotype, therapeutic resistance, poor prognosis of patients in multiple cancer types^8,9^.

Abnormal cell proliferation and apoptosis are always believed as major features of cancer. Earlier studies showed that the expression of MT1 significantly correlated with some clinicopathological features in HCC, such as tumor size^10^, Tumors, Nodes and Metastasis Classification (TNM) stage^11^, tumor grade^12^, which suggested MT1 subgroups seemingly play important roles in tumor growth. A study performed in thyroid cancer even showed that MT1G either suppresses proliferation, invasion or induces apoptosis^13^. However, the exerting functions of MT1G are not consistent in different cancer types. For examples, MT1G did not affect the proliferation and apoptosis, but promotes the differentiation and chemotherapy sensibility in colorectal cancer^14^. Before our study, MT1G was only proved to be downregulated to facilitate sorafenib resistance in HCC^15^. Nevertheless, the biological functions of MT1G in HCC have never been investigated. Consequently, we performed this study to investigate the effects of MT1G on the proliferation and apoptosis of HCC cells and its underlying mechanisms.

## Results

### Downregulation of MT1G is associated with poor prognosis of HCC patients

**Fig. 1:**
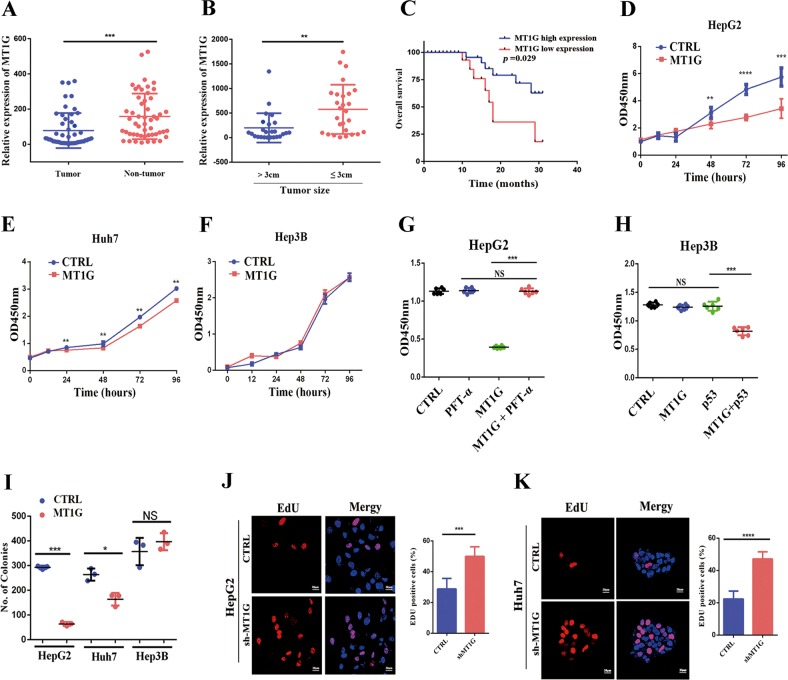
The evaluation of the clinical significance of MT1G and its effect on the proliferation of HCC cells. **a** RT-qPCR analysis of MT1G mRNA level in 52 human HCC tumor samples and their adjacent non-tumorous liver tissue samples. **b** Tumor size was inversely correlated with MT1G mRNA expression in HCC tissues. The mean expression value of all 52 cases was chosen as the cutoff value for separating the dataset into MT1G–low expression group and MT1G–high expression group. Quantitative analysis of MT1G expression in small HCC (diameter ≤ 3 cm) *vs* large HCC (diameter > 3 cm) at mRNA level. **c** Kaplan–Meier curves revealed an association of lower MT1G levels with a worse overall postoperative survival; **p < 0.01, ***p < 0.001, ****p < 0.0001. **d**–**f** Proliferation measured by CCK8 assay in HepG2 (**d**), Huh7 (**e**) and Hep3B (**f**) cell lines with or without MT1G overexpression. Data are presented as means ± SD. **p < 0.01, ***p < 0.001. **g**, **h** Proliferation was evaluated by CCK8 since p53 either was blocked by its inhibitor PFT-α (20 μM) in MT1G-overexpressed HepG2 cells (**g**) or was co-overexpressed with MT1G in Hep3B cells (**h**). **i** Statistical analysis for colony formation assay. Data are presented as means ± SD; *p < 0.05, ***p < 0.001, NS means non-significant. **j**, **k** Representative EdU labeling of HepG2 (**j**) and Huh7 cells (**k**) with MT1G knockdown; ***p < 0.001, ****p < 0.0001.

Before this study, we applied isobaric tags for relative and absolute quantitation (iTRAQ)-based proteomic strategy to analyze the proteome differences among small, medium, large and huge primary HCC tissues^16^. We finally identified and validated eight differentially expressed proteins, which significantly correlated to tumor size. Among them, MT1G was the one that changed most dramatically as the tumor grew larger. Thereby, in the present study, we wish to further explore the biological functions of MT1G in HCC cells. The results suggested that the expression of MT1G in HCC tissues was significantly downregulated compared to the paired non-tumor tissues (Fig. 1a, *p* = 0.0006, paired *t*-test), which was consistent with The Cancer Genome Atlas (TCGA) data (Supplementary Fig. S1A, *p* < 0.0001) and previous reports^16–18^. It is reported that 3 cm of tumor diameter is the most commonly used and widely accepted cut-off to analyze the clinical data in HCC^19–22^. Thus, we also select 3 cm as a threshold to divide the samples into large HCC (diameter > 3 cm) and small HCC (diameter ≤ 3 cm). According to the results, the expression of MT1G in large HCC was significantly less than that in small HCC (Fig. 1b, *p* = 0.002, unpaired *t*-test). These results suggested that MT1G might be involved in initialization and development of HCC.

To understand the significance of MT1G expression in HCC, we analyzed the association between MT1G mRNA levels and the clinical features of the HCC patients. The results showed the expression of MT1G significantly affected the tumor size (*p* = 0.005, Supplementary Table S1). The linear regression analysis also revealed a significant negative correlation between the tumor size and the expression of MT1G (*p* = 0.046, *R*^2^ = 0.0821, Supplementary Fig. S1B). The mean expression of MT1G was used as a threshold to separate the sample set into two groups (MT1G-high expression and MT1G-low expression), and Kaplan–Meier analysis revealed an association between lower MT1G expression levels and shorter overall survival time (*p* = 0.029, Fig. 1c). These findings suggested that the downregulation of MT1G may contribute to facilitate tumor growth of HCC.

### MT1G suppresses the proliferation of HCC

To elucidate the role of MT1G in HCC tumor growth, three HCC cell lines from different genetic background were used to investigate the effects of MT1G on the proliferation and apoptosis of HCC cells. CCK8 assays showed that the proliferation was significantly suppressed in both of MT1G-overexpressed HepG2 and Huh7 cells (Fig. 1d, e). However, MT1G did not affect the proliferation of Hep3B cells (Fig. 1f). Next, in the colony formation assay, a similar result was obtained. As shown in Fig. 1i, colonies formed in HepG2 and Huh7 cells with MT1G overexpression were significantly less than control cells (representative images are shown in Supplementary Fig. S1D). However, the quantities of colonies were similar in Hep3B cells with or without MT1G overexpression (Fig. 1i and Supplementary Fig. S1D). It has been reported that HepG2 cell line harbored a wild-type p53 (ref. ^23^), while a Y220C homozygous mutant p53 is harbored in Huh7 cell line (Supplementary Fig. S1F)^24^. For Y220C mutation of p53, it is reported that this mutation weakened the anti-tumoral ability of p53 through rapidly denaturing and aggregating p53 (refs. ^25,26^). Whereas, Hep3B cell line is a p53 natural deficiency cell line^27^. Consequently, we speculated that p53 signaling pathway seemingly was involved in the inhibition of proliferation induced by MT1G overexpression. To confirm our hypothesis, inhibitor PFT-α (20 μM)^28^ was applied to block the roles of p53 in HepG2 cells with MT1G overexpression. Meanwhile, wild type p53-Myc plasmid was co-transfected with MT1G-flag into Hep3B cells for 24 h to restore the function of p53. After the incubation for extra 48 h, the cell viability was detected with CCK8. The results showed that MT1G completely lost the inhibitory capacity on proliferation in HepG2 cells, since p53 was inactivated by PFT-*α* (Fig. 1g). Oppositely, MT1G exhibited the inhibitory capacity on proliferation in MT1G-overexpressed Hep3B cells that re-transfected with p53-Myc plasmid (Fig. 1h). Additionally, MT1G was knocked down by specific shRNA in HepG2 cells and Huh7 cells (Supplementary Fig. S1E). And EdU assay and CCK8 assay showed that MT1G knockdown accelerated the proliferation of HCC cells with p53 background (Fig. 1j, k and Supplementary Fig. S1C). Altogether, these results proposed and confirmed a notion that MT1G inhibited proliferation of HCC cells in a p53-dependent manner.

### MT1G promotes the apoptosis of HCC

**Fig. 2:**
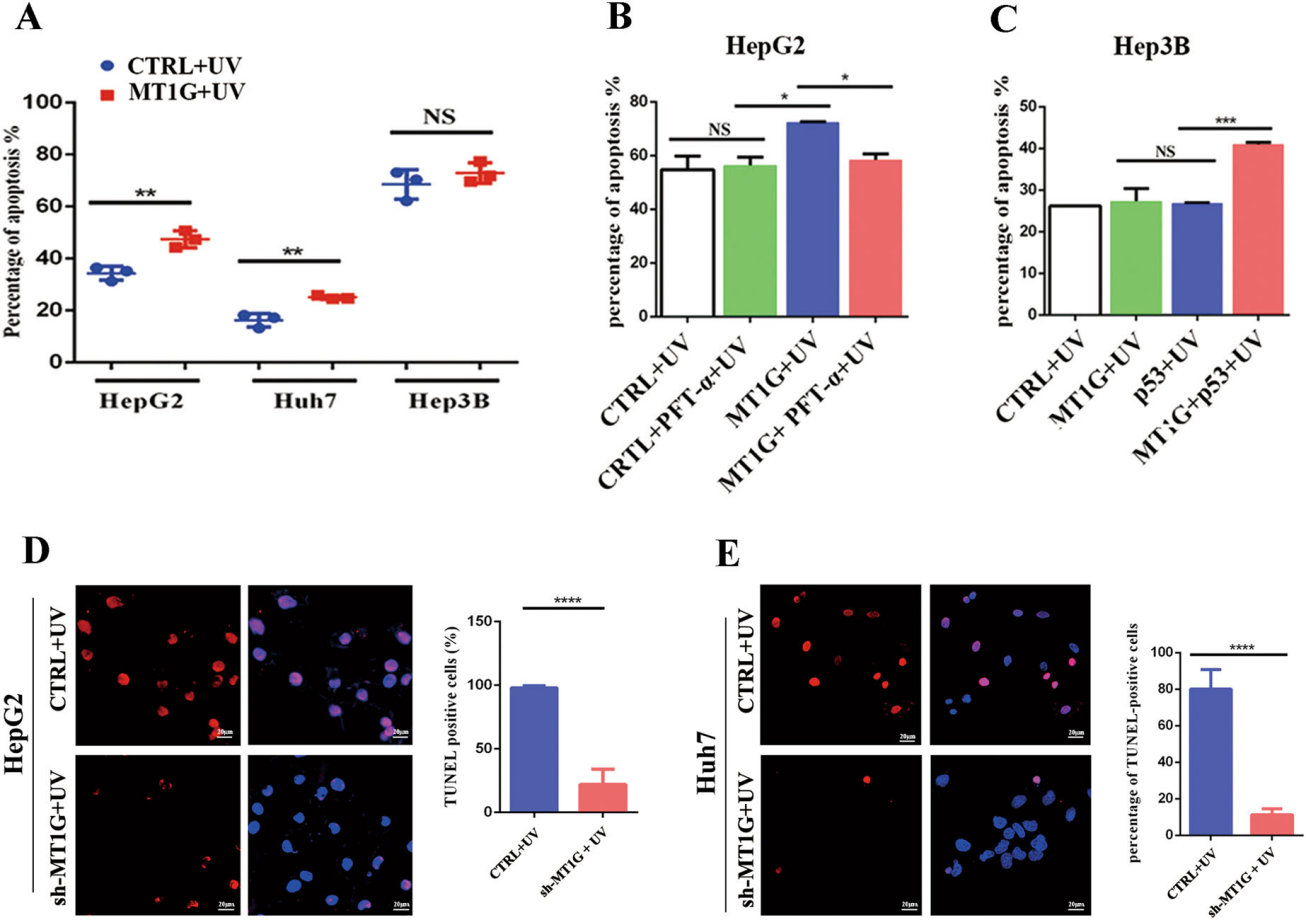
MT1G enhances the apoptosis of HCC cells. **a** Statistical analysis of the apoptosis measured by Annexin V/propidium iodide in HepG2, Huh7 and Hep3B cells with or without MT1G overexpression. Data are presented as means ± SD; **p < 0.01, NS means non-significant. **b**, **c** Statistical analysis of the apoptosis measured by Annexin V/propidium iodide, since p53 either was blocked by its inhibitor PFT-α (20 μM) in MT1G-overexpressed HepG2 cells (**b**) or was co-overexpressed with MT1G in Hep3B cells (**c**). Data are presented as means ± SD; *p < 0.05, ***p < 0.001, NS means non-significant. **d**, **e** Representative TUNEL labeling of HepG2 (**d**) and Huh7 (**e**) cells with MT1G knockdown; ****p < 0.0001.

To verify whether MT1G is involved in the regulation of p53-dependent apoptosis, and because UV irradiation-induced cell apoptosis is depending on p53 signaling pathway^29^, we evaluated the effects of MT1G on apoptosis induced by UV irradiation in HCC cell lines. The results were as per our expectation, MT1G effectively promoted apoptosis induced by UV irradiation for 16.26% in HepG2 cells (Fig. 2a) and the apoptosis in Huh7 cells was significantly enhanced for 12.5% (Fig. 2a). However, MT1G did not exert the regulatory effect in Hep3B cells (Fig. 2a). The representative images were shown in Supplementary Fig. S2A, B and C. These observations were further confirmed by invalidating or restoring the function of p53. The PFT-*α* (20 μM) and p53-Myc plasmid (2 μg) were supplemented or re-transfected into MT1G-overexpressed HepG2 or Hep3B cells, respectively, as performed in proliferation assay. Similarly, the regulatory capacity of MT1G on apoptosis disappeared or arose, respectively, in MT1G-overexpressed HepG2 cells (Fig. 2b) and Hep3B cells (Fig. 2c). The representative images of these verified assays are shown in Supplementary Fig. S2D, E. Furthermore, TUNEL assay suggested that MT1G knockdown significantly inhibited apoptosis induced by UV irradiation in HepG2 and Huh7 cells (Fig. 2d, e). Overall, our results proposed and confirmed a notion that MT1G promoted the apoptosis of HCC in p53-dependent manner.

### MT1G acts as a tumor suppressor through modulating p53 pathway

**Fig. 3:**
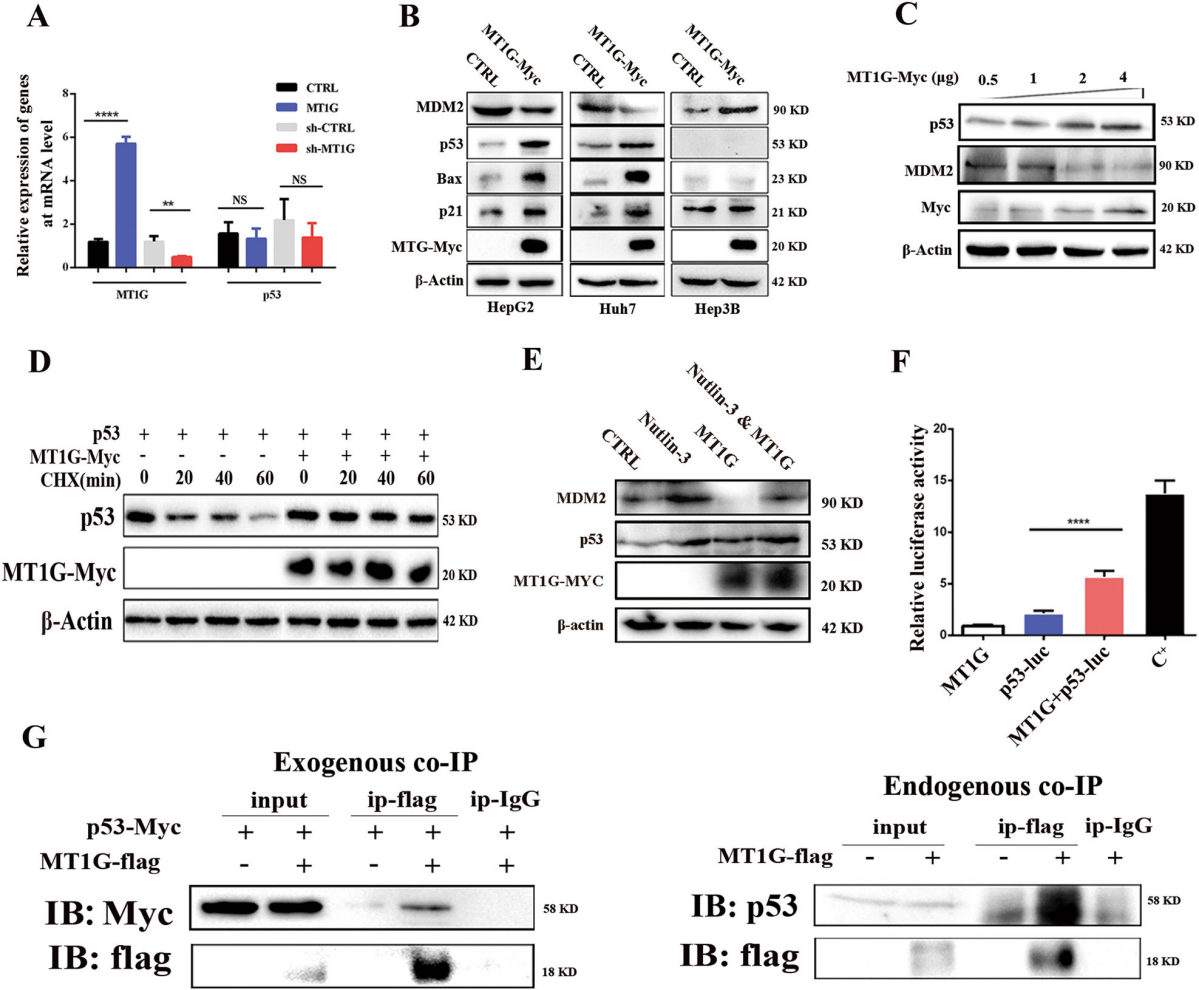
MT1G exerts its anti-proliferation and pro-apoptosis *via* the p53 signaling pathway. **a** Relative expression of p53 was investigated by qRT-PCR in HepG2 cells with MT1G overexpression; **p < 0.01, ****p < 0.0001, NS means non-significant. **b** The protein levels of MDM2, p53, Bax and p21 was evaluated in HepG2, Huh7 and Hep3B cells with or without MT1G overexpression. **c** The protein level of p53 and MDM2 were, respectively, increased or decreased by MT1G in a dose-dependent manner. **d** MT1G prolongs the half-life of p53. MT1G-flag and p53-Myc were transfected into 293T cells and then treated with CHX (100 μg/ml) for the indicated times. **e** Western blot analysis of p53, Mdm2, MT1G and β-actin in MT1G overexpressed 293T cells pre-treated with 50 nM of Nutlin-3 for 24 h. **f** MT1G-induced p53 transcriptional activity. The p53-Luciferase reporter and β-galactosidase (β-gal) gene expression vectors, together with p53, MT1G expression vectors were transfected into 293T cells as indicated. Reporter gene activity was determined and normalized in relation to the co-transfected β-gal activity. The bars represent the mean ± SD from three independent experiments. C^+^ means positive control in which the cells were co-transfected with p53-Myc plasmid and p53-luciferase reporter. **g** Co-IP analysis of MT1G and p53. For the exogeneous assay, 293T cells were transfected with MT1G-flag and p53-Myc plasmids (left panel). For the endogenous assay, HepG2 cells were transfected with MT1G-flag plasmid (right panel). Cell lysates were immunoprecipitated with anti-flag antibody. The immunoprecipitates and cell lysates were then analyzed by Western blotting separately using anti-Myc (for exogeneous p53), anti-p53 antibody and anti-flag (for exogeneous MT1G). There is no non-specific reaction between MT1G-flag and IgG.

Firstly, the expression of p53 at mRNA level was examined with quantitative real-time polymerase chain reaction (RT-qPCR). The results showed that the expression of p53 was not regulated by MT1G at the mRNA level (Fig. 3a), which is consistent with the TCGA data (Supplementary Fig. S3A). However, overexpression of MT1G in HepG2 and Huh7 cell lines caused significant upregulation of p53 protein level and its downstream factors, including p21 and Bax rather than the MDM2, which was downregulated (Fig. 3b). An increasing evidence showed that p21 was a cyclin-dependent kinase inhibitor (CKI) that mediated cell cycle arrest^28^; Bax is identified as pro-apoptotic member of the Bcl-2 protein family^30^; and MDM2 is a major ubiquitination factor of p53 (ref. ^31^). However, overexpression of MT1G failed to alter the expression of p21 and Bax in the p53-null Hep3B cells, although the expression of MDM2 was upregulated (Fig. 3b). Therefore, we believe that MT1G increases p53 protein level by reducing MDM2-mediated degradation of p53. To confirm this notion, we transfected gradient concentrations of MT1G overexpression plasmids to 293T cells and investigated the expressions of MDM2 and p53. The results showed that MT1G regulated the expression of p53 and MDM2 in a dose-dependent manner (Fig. 3c and Supplementary Fig. S3B). To evaluate the effect of MT1G on p53 protein stability, 293T cells were treated with cycloheximide (CHX), an inhibitor of protein synthesis. Western blot analysis showed that MT1G significantly prolonged the half-life of p53 resulting in an indirect increase of p53 protein levels (Fig. 3d and Supplementary Fig. S3C). To prove the notion that MDM2 is involved in the degradation of p53 in this study, MDM2 antagonist, nutlin-3, was applied to block the interaction between MDM2 and p53. The results suggested that the expression of p53 was increased by incubating with nutlin-3 and/or by MT1G overexpression (Fig. 3e).

**Fig. 4:**
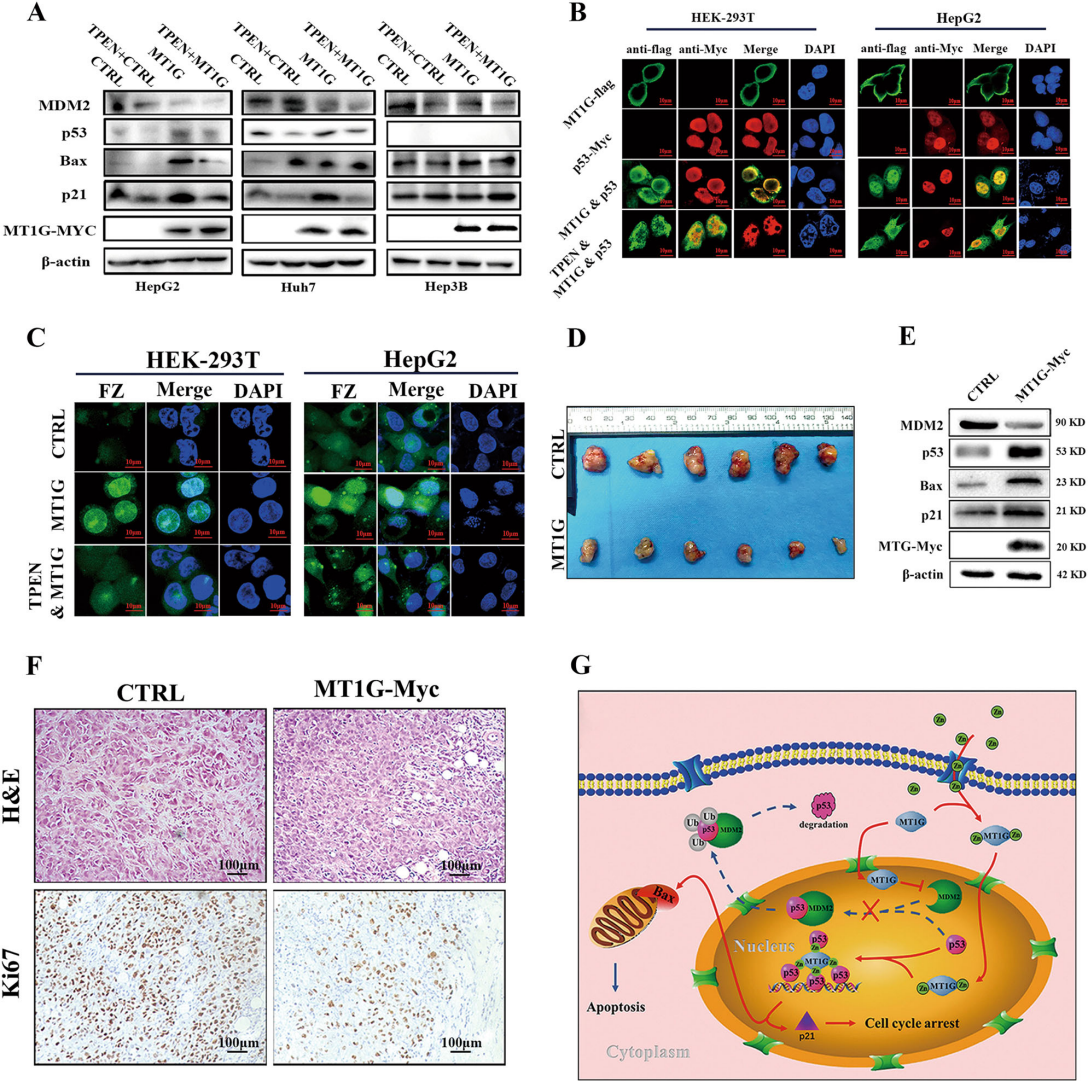
The assessment of the role of zinc ions and the evaluation of biological functions of MT1G in vivo. **a** The presence of zinc chelator attenuated the regulation roles of MT1G to p53, p21 and Bax in p53 background cells. **b** Subcellular localization of MT1G and p53 in 293T or HepG2 cells. Both MT1G-flag and p53-Myc plasmids were transfected into cells as indicated. For the inhibitory assay, MT1G-overexpressed cells were pre-treated with 100 μM of TPEN for 24 h. Cells were immunostained for detecting MT1G by flag antibody followed by Alexa-488 conjugated secondary antibody and for detecting p53 by anti-p53 antibody followed by Alexa-546 conjugated secondary antibody. Nucleus were stained by DAPI. Stained cells were visualized with confocal microscope (magnification: 630 folds). **c** Subcellular localization (at nucleus) of zinc ions visualized by fluorescence imaging. MT1G overexpression plasmid or control plasmid were transfected into HepG2 or 293T cells. Afterward, FluoZin-3-AM in PBS was used to incubate the transfected cells for 30 min. In the inhibitory assay, 0.1 μM of TPEN was supplemented to incubate with the MT1G transfected cells for 30 min prior to Fluor Zin-3-AM supplementation. Afterward, the cells were washed and visualized by the confocal microscope (magnification: 630 folds). **d** Macrograph of tumors harvested from nude mouse inoculated by HepG2 cells with or without MT1G overexpression. **e** Western blot analysis of the expression of MDM2, p53, Bax, p21 and MT1G-Myc protein in HepG2-MT1G tumors and control tumors. **f** Representative photographs of H&E staining and Ki-67 specific staining in tumor tissue (magnification: 200 folds). **g** Hypothetical model depicting the proposed mechanism by which MT1G regulates proliferation and apoptosis of HCC cells through p53 pathway.

On the other hand, since p21 and Bax as the major transcriptional targets of p53, we speculated that MT1G indirectly lead to p21 and Bax upregulation by simply enhancing the transcriptional activity of p53. Consequently, p53-luciferase reporter system was applied to examine the activity of p53. As shown in Fig. 3f, MT1G significantly induced the activity of the p53 reporter gene. Additionally, it has been reported that p53 protein was a zinc-binding transcription factor, and MT1G was a key zinc chelate protein. Thus, we speculated that MT1G promoted the transcriptional activity by interacting with or donating appropriate zinc ions to p53. We further investigated this possibility using co-immunoprecipitation (co-IP) assays. To our astonishment, p53 was detected in the MT1G immunoprecipiates (Fig. 3g). Moreover, *N*,*N*,*N*′,*N*′-tetrakis (2-pyridylmethyl) ethylenediamine (TPEN) as a zinc chelator was applied to treat the HepG2, Huh7 and Hep3B cells. As shown in Fig. 4a, chelating zinc with TPEN attenuated the induction of p53, p21 by MT1G in HepG2 and Huh7 cells. In addition, confocal microscopy observation showed that MT1G and p53 were co-located in the nucleus of 293T and HepG2 cells (Fig. 4b). However, the presence of TPEN caused less MT1G translocation into nucleus (Fig. 4b). Next, we evaluated intracellular zinc ion levels with FluoZin-3-AM (FZ). As expected, MT1G not only increased the concentration of intracellular zinc ions, but also enriched it in the nucleus (Fig. 4c). Nevertheless, this enrichment was attenuated by TPEN supplementation. Altogether, these results suggested that MT1G could enhance p53 transcriptional activity through interacting with and providing appropriate zinc ions to p53.

### MT1G suppresses HCC progression in vivo

Next, an in vivo study was performed to verify that MT1G suppresses HCC tumor growth. After transplantation for 6 weeks, tumors were successfully formed in nude mice of both groups. However, the tumors of MT1G-overexpressed HepG2 cells grew significantly slower than the control group (*n* = 6, Fig. 4d and Supplementary Fig. S3D). To correlate the biological responses with the mechanisms identified in vitro, MDM2, p53, p21 and Bax protein levels were assessed by western blot. As shown in Fig. 4e, the overexpression of MT1G upregulated the protein levels of p53, p21 and Bax rather than MDM2, which is consistent with our results in vitro. Meanwhile, less Ki-67 staining was observed in MT1G-overexpressed tumor tissues (Fig. 4f). These data indicate that MT1G is able to suppress HCC tumor growth in nude mice.

## Discussion

High recurrence rate, rapid growth and drug resistance are the main reasons causing HCC difficult to cure. Many target drugs have been developed to inhibit tumor growth. However, only a few target drugs have been approved for systemic therapy of advanced HCC patients and their efficacy are still unsatisfactory. Hence, regulation mechanism of HCC growth remains to be elucidated. In our previous study, MT1G, but not other MTs, was screened out, which is negatively correlated to tumor size^16^. Thereby, the effects of MT1G on proliferation and apoptosis of HCC cells were further investigated in the present study. Our results firstly confirmed our previous observation: MT1G is significantly downregulated in tumoral tissue and the downregulatory extent of MT1G is significantly associated with tumor size and poor prognosis (Fig. 1a, c and Supplementary Table S1). Afterward, we found that MT1G inhibited proliferation and enhanced apoptosis of HCC cells. Additionally, we also identified the p53 signaling pathway as a single target that was responsible for the anti-proliferative and pro-apoptosis functions of MT1G. The mechanism study further confirms a notion that MT1G suppresses HCC tumor growth through regulating the expression and transcriptional activity of p53 (Fig. 4g).

MTs consist of 16 functional MT isoforms that are divided into four subgroups (MT1, 2, 3 and 4). Noteworthily, increasing reports showed that MTs were involved in many physiological and pathophysiological processes of cancer, such as apoptosis, proliferation, metastasis and drug resistance^32–35^. It has been reported that MTs behaved as cancerogenic factors or tumor suppressors depending on the different cancer types. While previous reports showed that expression of MT1 subgroup was downregulated due to promoter methylation in HCC^36,37^. Some other isoforms of MT1 were identified as tumor suppressors such as MT1M and MT1H in HCC^32,38^. Moreover, double deletion of MT1 and MT2 promotes diethylnitrosamine-induced hepatocarcinogenesis in mice^39^. All these reports seemingly indicated that MT1G was a candidate tumor suppressor in HCC, however, which has never been investigated before our study.

In our phenotype study, MT1G obviously inhibited the proliferation and enhanced the UV-induced apoptosis in the cells with p53 activity. However, MT1G completely failed to induce the phenotypic change in Hep3B cells with p53 congenital deletion. These results potentially indicate that p53 signaling pathway is primarily involved in the regulation of MT1G to proliferation and apoptosis in HCC cells. In verification assays, MT1G completely lost the regulatory capacity in HepG2 cells, since PFT-α was supplemented to culture the cells with MT1G overexpression. Together with the verification assay, these results provided abundant evidences to confirm that p53 signaling pathway, as a single target, plays critical roles in the process of MT1G suppressing HCC. Our observation is incompletely consistent with previous study performed in other cancer types, in which NF-κB signaling pathway, ERK1/2 signaling pathway and AKT signaling pathway are also involved in the regulation of MTs to proliferation and apoptosis of cancer cells, besides p53 signaling pathway^13,40,41^. The reasons are probably attributed to the different cancer types, tumor progression stage and even MTs isoforms.

According to our results, two underlying mechanisms are used to explain how MT1G exerted its biological functions. One is that MT1G increases p53 protein level by inhibiting the expression of MDM2. It has been well accepted that misregulation of the p53-mdm2 loop usually lead to mdm2 stabilization and p53 degradation in homeostatic cancer cells^42^. However, MT1G overexpression dramatically inhibits the expression of MDM2 (Fig. 3b) leading to enhance the stability of p53. Consistently, the phosphorylation of AKT was obviously reduced by MT1G (Supplementary Fig. S3E), whose phosphorylation has been demonstrated to enhance the stability and activity of MDM2 (ref. ^31^). The other underlying mechanism is that MT1G enhances the transcriptional activity of p53 through interacting with or providing appropriate zinc ions to p53. In this study, the enhancement of p53 transcriptional activity was firstly proved in cells with MT1G overexpression. Afterward, the interaction between MT1G and p53 was discovered for the first time (Fig. 3g). And the confocal images further confirmed that MT1G, p53 and zinc were colocalized at the nucleus (Fig. 4b, c). It is well known that zinc is essential to keep the stability of p53 tetramer and its transcriptional activity^7,43^, and MT1G is broadly considered as a zinc chelator protein. Hence, we proposed that MT1G as a vehicle to provide appropriate zinc ions to p53 and probably binds to it through zinc ions. The subsequently inhibitory assay ideally supports this speculation. Chelating intracellular zinc with TPEN attenuated the effects of MT1G to p53 and its downstream factors in HepG2 and Huh7 cells (Fig. 4a).

Our study highlights the crucial role of MT1G in suppressing the progression of human HCC by inhibiting cell proliferation and enhancing apoptosis. Our data showed that modulation of MT1G could represent a future therapeutic strategy for the treatment of HCC.

## Materials and methods

### Human tissue specimens

The fresh frozen tumor samples were randomly collected from HCC patients at Mengchao Hepatobiliary Hospital of Fujian Medical University between 2013 and 2015. Clinical and pathologic diagnosis of HCC met the criteria of the American Association for the Study of Liver Diseases. Our research was approved by Mengchao Hepatobiliary Hospital Medical Ethics Committee of Fujian Medical University. Informed consent was received from each participant before surgery in this study.

### Cell culture and reagents

The human hepatoma cell lines HepG2, Hep3B and the human embryonic kidney cell line HEK293T were purchased from ATCC cell bank in 2014. The human hepatoma cell line Huh7 was purchased from the cell bank of the Shanghai Institute of Cell Biology (Shanghai, China) in 2017. HepG2 and Hep3B were maintained in Minimum Essential Medium (MEM, Gibco, Thermo, USA). Huh7 and 293T were maintained in Dulbecco’s modified Eagle’s medium (DMEM, Gibco, Thermo, USA). The complete cell culture mediums were supplied with 10% fetal bovine serum (FBS, Excel, Australia), 100 U/ml penicillin and 100 μg/ml streptomycin. Cells were incubated in a CO_2_ incubator at 37 °C, with 5% CO_2_. All the cell lines were used for less than 25 passages from acquisition to discard. Pifithrin-α (PFT-α) was purchased from Santa Cruz Biotechnology (USA), nutlin-3 was from Selleck Chemicals (USA) and TPEN was from Sigma-Aldrich Inc. (USA). For the inhibitory assay, PFT-a (20 μM), nutlin-3 (50 nM) and TPEN (100 nM) were dissolved in DMSO, Milli-Q sterile water or ethanol, respectively, and applied to pretreat the cells.

### Plasmids and cell transfection

The full-length human MT1G sequence (GenBank number: NM_001301267) was synthesized by GENEWIZ (China) and cloned into a lentiviral vector to construct an MT1G overexpression plasmid, SWP-MT1G-Myc. To knock down MT1G, human short hairpin RNA (sequence: GATCCCTGCAAGAAGAGCTGCTGCTCCTTCCTGTCAGAGAGCAGCAGC-TCTTCTTGCAGTTTTTG) was synthesized by Sangon Biotech (China), followed by cloning into lentiviral vector pGreen-shMT1G-puro. The lentiviruses were packaged as previously described^44^. p53-Myc plasmid with a wild-type p53 sequence in previous published report^29^ was provided by professor Wu as a kind gift. For a stable cell transfection, HepG2, Huh7 and Hep3B cells were infected by lentivirus in the presence of 2 μg/ml polybrene (Santa Cruz Biotechnology, USA), followed by selecting with 2 μg/ml puromycin for 2 weeks. For transiently co-overexpression study, the sequence of MT1G was cloned into vector pcDNA3.1-flag vector. To knock down MT1G in cells, the vector pGreen-shMT1G-puro was transiently transfected into HepG2 cells and Huh7 cells with lipofectamine 3000 (Thermo, USA) according to the manufacturer’s instructions.

### Cell proliferation assay

For CCK8 assay, cells were seeded into 96-well plates at a density of 3 × 10^3^ cells per well, and were incubated in saturated humidity with 5% CO_2_ at 37 °C. After the indicated incubation time, CCK8 (Dojindo Molecular Technologies, Inc., Japan) was added to each well and further incubated for 2 h at 37 °C. The optical density (OD) was measured at wavelengths of 450 nm and using a V Max kinetic microplate reader at indicated time periods. In the verification assay, 20 μM of PFT-α was supplemented to culture MT1G-overexpressed HepG2 cells for 48 h. Or, the Hep3B cells were co-transfected by p53-Myc and MT1G-flag plasmids and incubated for 24 h. Then, the transfected cells were seeded into 96-well plates at a density of 3 × 10^3^ cells per well, and further incubated for extra 48 h. Afterward, the cell viability was evaluated with CCK8 kit. Parallelly, HCC cells with MT1G knockdown were plated into 96-well plates at a density of 3 × 10^3^ cells per well. After 48 h of incubation, the cell viability was evaluated with CCK8 kit, as mentioned above. These experiments were repeated independently at least three times. For EdU incorporation assay, 5 × 10^4^ modified cells were seeded in 35-mm glass-bottom dish (ibidi, Germany). After incubation for 24 h, the cells were labeled with EdU labeling kit (Sangon Biotech, China) following the manufacturer’s instruction. Subsequently, the cells were visualized using a confocal microscope (Zeiss LSM780, X400). The ratio of the number of EdU-positive cells (red cells) to the total number of DAPI-positive cells (blue cells) was applied to assess the EdU incorporation rate.

### Cell colony formation assay

Cells with or without MT1G overexpression were seeded into 6-well plate at a density of 3 × 10^3^ cells per well and allowed to grow for 2 weeks. Colonies were visualized by crystal violet staining later, as previously described^45^. This experiment was repeated independently at least three times.

### Cell apoptosis assay

Apoptosis induction were performed as previously described^29^ with slight modification. Briefly, the cells were irradiated with UV (50 J/m^2^) for 5 min and then were incubated for 6 h. Subsequently, the irradiated cells were harvested for apoptosis analysis using Annexin V/PI staining as previous described^46^. In the inhibitory or restoring assay, MT1G-overexpressed HepG2 cells were pre-treated with PFT-α (20 μM) for 1 h. Afterward, the apoptosis induction and analysis were performed as before. Accordingly, Hep3B cells was co-transfected by p53-Myc and MT1G-flag plasmids and incubated for 24 h. Then the apoptosis was induced and analyzed in the transfected cells as previously reported. The cell apoptosis was calculated with the percentage of apoptosis cells in the upper right gate plus that in the lower right gate. This experiment was repeated independently at least twice. The apoptosis of HCC cells with MT1G knockdown was assessed with the TUNEL system. The modified cells were seeded in 35-mm glass-bottom dish (ibidi, Germany) at a density of 5 × 10^4^ cells per well. Then, the cells were labeled with TUNEL apoptosis assay kit (Beyotime, China) according to the manufacturer’s instructions. Afterward, the cells were visualized under a confocal microscope (Zeiss LSM780, magnification: 400 folds). The percentage of apoptosis cells was calculated with the ratio of the number of TUNEL-positive cells (red cells) to the total number of DAPI-positive cells (blue cells).

### RT-qPCR assay

Total RNA isolation and RT-qPCR were carried out using described procedures^16^. The human 18S rRNA was chosen as reference gene to normalize data and the relative expression was calculated according to the formula: 2^−ΔΔC(t)^. Complementary DNA from various cell samples was amplified with specific primers (human MT1G primer pair: 5′-AAAGGGGCATCGGAGAAGTG-3′ and 5′-GCAAAGGGGTCA-AGATTGTAGC-3′, human p53 primer pair: 5′-AACTGCGGGACGAGACAGA-3′ and 5′- AGCTTCAAGAGCGACAAGTTTT-3′, human 18S rRNA primer pair: 5′-AGAAACGGCT-ACCACATCCA-3′ and 5′-CACCAGACT-TGCCCTCCA-3′). These experiments were repeated independently at least three times.

### Western blot

Western blot was used to analyze protein expression as described^16^. Briefly, 50 μg of cell lysate was separated by SDS-PAGE and transferred onto the NC membranes (PALL Corporation, USA). After blocking, the membranes were probed with primary antibodies: MT1G (1/1000 dilution, Omnimab, #OM263051, USA), p53 (1/1000 dilution, Cell Signaling Technology, #2527s, USA), MDM2 (1/1000 dilution, Cell signaling Technology, # 86934, USA), p21 and Bax (1/500, Santa Cruz Biotechnology, # sc-136020, # sc-20067, USA), and beta-actin (1/2000 dilution, TransGen Biotech, #HC201-01, China). The blots were then incubated with horseradish peroxidase-conjugated secondary antibodies (TransGen Biotech, #HS101-01) and visualized by enhanced chemiluminescence (Thermo, USA). Quantification of blot was analyzed by densitometry and normalized by β-actin. Western blot analysis was repeated independently at least twice.

### Luciferase reporter assay

The luciferase reporter assay was performed as previously described^27^. This experiment was repeated independently at least three times.

### co-IP assay

MT1G DNA sequence was sub-cloned to pcDNA3.1-flag vector for this research. All immunoprecipitation procedures were carried out at 4 °C as previous described^27^. This experiment was repeated independently twice.

### Immunofluorescence staining

For MT1G and p53 location assay, the cells were fixed in 4% paraformaldehyde. After blocking, the cells were probed with anti-Flag antibody (1/500 dilution, TransGen biotech, # HT201-02, for MT1G staining) and anti-Myc antibody (1/500 dilution, Cell Signaling Technology, # 2278, for p53 staining) followed by incubation with Alexa Fluor 488 or Alexa Fluor 546-conjugated secondary antibody (Thermo Fisher scientific, # A-11029, # A10040, 1/500 dilution). Cells were further incubated with DAPI (Sigma, 50 μg/ml) to stain the nuclei. For visualization of intracellular zinc level, the cell-permeable zinc-specific indicator FZ (Invitrogen) was used. 293T cells were incubated with 2 μmol/l FZ for 30 min. After washing, the cells were incubated for further 30 min to allow for the intracellular cleavage and activation of the fluorophore. Finally, the stained cells were visualized by a confocal microscope (Zeiss, LSM780).

### In vivo assays for tumor growth

In this study, 12 BALB/c male nude mice with 6–8 weeks old were randomly divided into two groups. To generate murine subcutaneous tumors, 5 × 10^6^ HepG2 cells with or without MT1G overexpression in 200 μl of phosphate-buffered saline were mixed with equal volume of Matrigel (BD Biosciences, USA), prior to subcutaneous injection in the right of the dorsal midline of nude mice (*n* = 6). Eight weeks later, the animals were sacrificed. The tumor size was calculated according to the formula: volume (mm^3^) = 1/2 × (length × width^2^) (ref. ^47^). All animal experiments were performed under the guidance of Fuzhou General hospital Committee for Use and Care of Laboratory Animals and approved by the animal experimentation ethics committee.

### Immunohistochemistry assay

Paraffin-embedded tissue samples were serially sectioned and immunohistochemically examined using an immunohistochemical staining kit (Maixin Bio, China), according to the manufacturer’s instructions, with antibodies against Ki-67 (Maixin Bio, China).

### Statistical analysis

Prism statistical software (GraphPad v6.01, CA) and SPSS statistics (v19.0, SPSS Inc., USA) were employed for data analysis. Data are expressed as means ± SD of three independent experiments. Only the comparison of MT1G expression in tumor tissues and non-tumor tissues was performed with paired Student *t*-tests. Other comparisons used unpaired Student *t*-tests to analyze. *p* Value < 0.05 was considered significant. Homogeneity of variance test has been performed (*p* > 0.05) prior to *t*-test.

## Supplementary information

Supplementary Table S1

Supplementary Figure S1

Supplementary Figure S2

Supplementary Figure S3
