# Supplementary material for: MT1G serves as a tumor suppressor in hepatocellular carcinoma by interacting with p53
Source: Oncogenesis. 2019 Nov 15;8(12):67. doi: 10.1038/s41389-019-0176-5 (PMC6858331; doi:10.1038/s41389-019-0176-5)
Supplement: Supplementary file 1 — Supplementary Table S1 [file 41389_2019_176_MOESM1_ESM.docx]

**Table S1**

| Clinicopathological  variable | MT1G expression | | *p* Value |
| --- | --- | --- | --- |
|  | Low (n=27) | High (n=25) |  |
| Gender |  |  | 1.000 |
| Male | 24 | 22 |  |
| Female | 3 | 3 |  |
| Age, years |  |  | 0.052 |
| <55 | 10 | 16 |  |
| ≥55 | 17 | 9 |  |
| AFP, ng/mL |  |  |  |
| <400 | 20 | 23 | 0.143 |
| ≥400 | 7 | 2 |  |
| Maximal tumor size, cm |  |  |  |
| <3 | 9 | 18 | **0.005^*^** |
| ≥3 | 18 | 7 |  |
| Tumor number |  |  |  |
| Single | 24 | 23 | 1.000 |
| Multiple | 3 | 2 |  |
| Microvascular invasion |  |  |  |
| Absent | 19 | 15 | 0.432 |
| Present | 8 | 10 |  |
| TNM stage |  |  |  |
| I–II | 24 | 23 | 1.000 |
| III–IV | 3 | 2 |  |
| HBV |  |  |  |
| Absent | 6 | 2 | 0.252 |
| Present | 21 | 23 |  |
| Cirrhosis |  |  |  |
| Absent | 2 | 1 | 1.000 |
| Present | 25 | 24 |  |

Abbreviation: AFP, alpha-fetoprotein.

**p* < 0.05.
