# Supplementary figures and images for: MT1G serves as a tumor suppressor in hepatocellular carcinoma by interacting with p53
Source: Oncogenesis. 2019 Nov 15;8(12):67. doi: 10.1038/s41389-019-0176-5 (PMC6858331; doi:10.1038/s41389-019-0176-5)

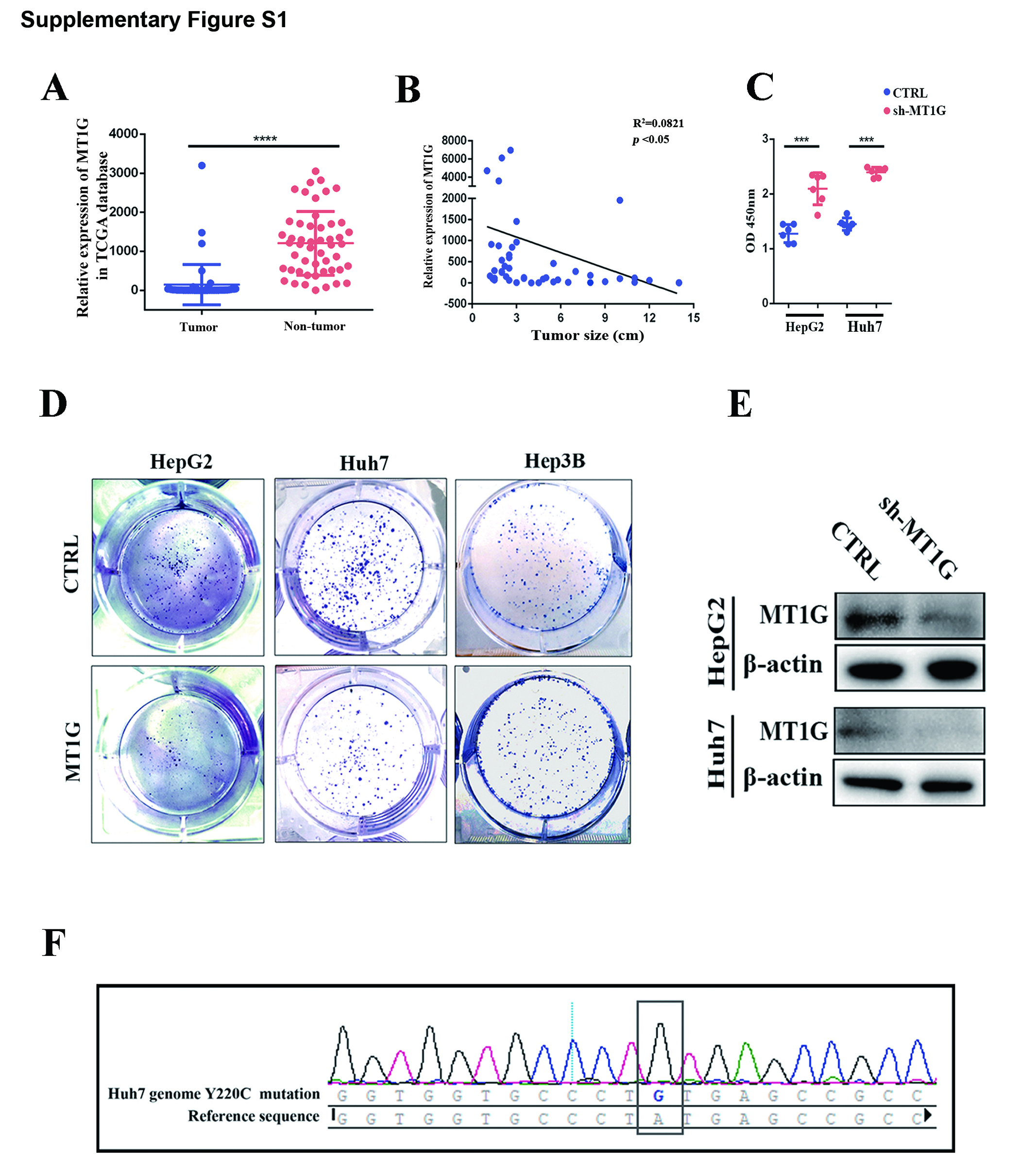

Supplement: Supplementary file 2 — Supplementary Figure S1 [file 41389_2019_176_MOESM2_ESM.jpg]

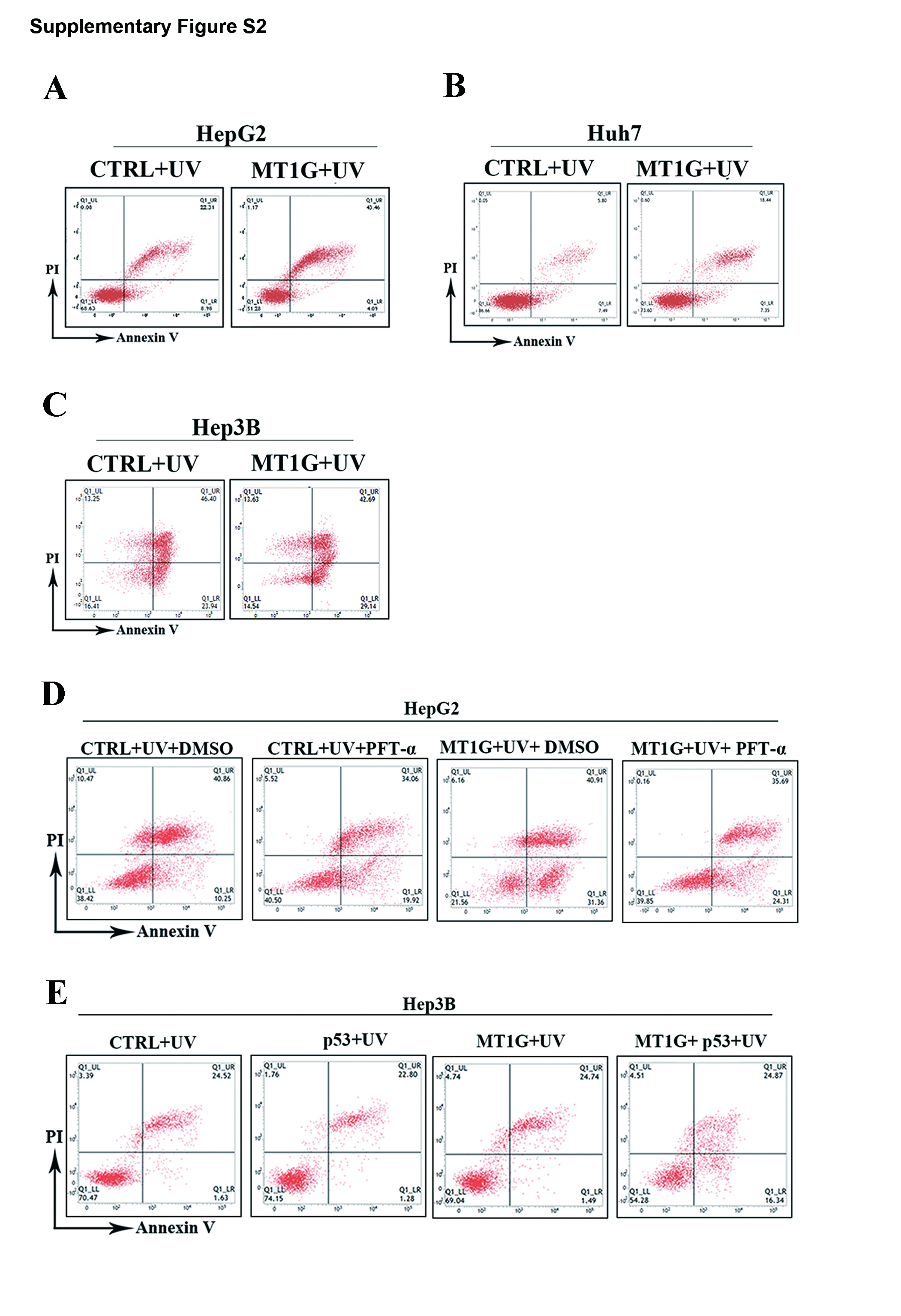

Supplement: Supplementary file 3 — Supplementary Figure S2 [file 41389_2019_176_MOESM3_ESM.jpg]

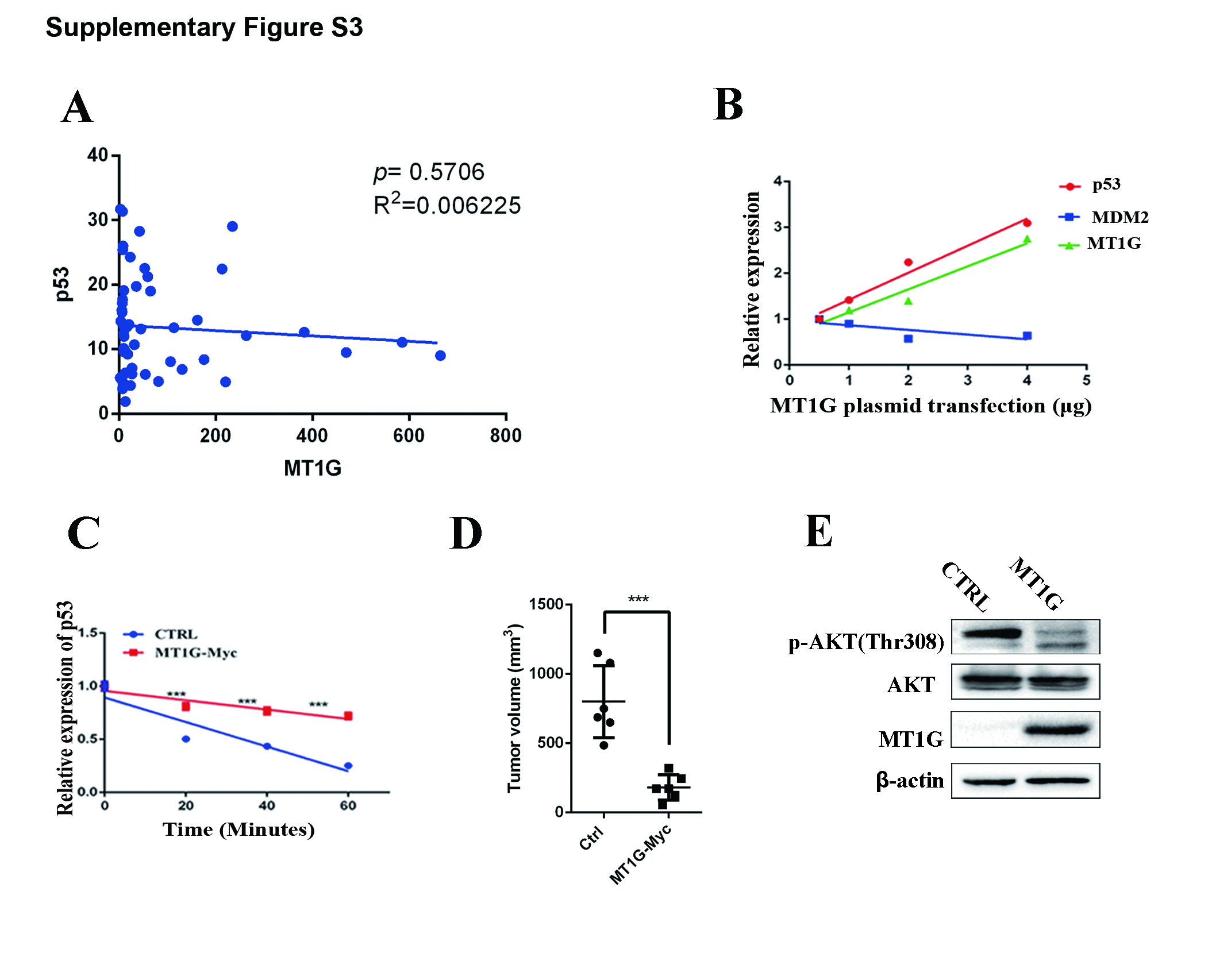

Supplement: Supplementary file 4 — Supplementary Figure S3 [file 41389_2019_176_MOESM4_ESM.jpg]
